# Adjuvant Ovarian Function Suppression in Premenopausal Hormone Receptor–Positive Breast Cancer

**DOI:** 10.1001/jamanetworkopen.2024.2082

**Published:** 2024-03-13

**Authors:** Robert B. Basmadjian, Sasha Lupichuk, Yuan Xu, May Lynn Quan, Winson Y. Cheung, Darren R. Brenner

**Affiliations:** 1 Department of Community Health Sciences, Foothills Medical Centre, University of Calgary, Calgary, Alberta, Canada; 2 Department of Oncology, Tom Baker Cancer Centre, University of Calgary, Calgary, Alberta, Canada; 3 Department of Surgery, Foothills Medical Centre, University of Calgary, Calgary, Alberta, Canada

## Abstract

**Importance:**

Few oncology studies have assessed the effectiveness of adjuvant ovarian function suppression (OFS) in observational settings for premenopausal hormone receptor–positive breast cancer. Target trial emulation is increasingly used for estimating treatment outcomes in observational cohorts.

**Objectives:**

To describe hormone therapy and OFS treatment patterns (aim 1), examine the association between adding OFS to tamoxifen (TAM) or aromatase inhibitor (AI) and survival (aim 2), and examine the association between duration of hormone treatment (TAM or AI) plus OFS (H-OFS) and survival (aim 3).

**Design, Setting, and Participants:**

This population-based cohort study included all premenopausal, early-stage breast cancer diagnoses between 2010 and 2020 in Alberta, Canada. Target trial emulation was conducted. Eligibility criteria were directly modeled after the Suppression of Ovarian Function Trial (SOFT) and Tamoxifen and Exemestane Trial (TEXT). Participants were followed up for a maximum of 5 years. Data were analyzed from July 2022 through March 2023.

**Exposures:**

For aim 2, exposures were receiving the following baseline treatments for 2 years: AI + OFS (AI-OFS), TAM + OFS (T-OFS), and TAM alone. For aim 3, exposures were a 2-year or greater and a less than 2-year duration of H-OFS.

**Main Outcomes and Measures:**

Recurrence-free survival was the primary outcome of interest. Marginal structural Cox models with inverse probability treatment and censoring weights were used to estimate hazard ratios (HRs), adjusted for baseline and time-varying confounding variables.

**Results:**

Among 3434 female patients with premenopausal, early-stage breast cancer diagnoses (median [IQR] age, 45 [40-48] years), 2647 individuals satisfied SOFT and TEXT eligibility criteria. There were 2260 patients who initiated TAM, 232 patients who initiated T-OFS, and 155 patients who initiated AI-OFS; 192 patients received H-OFS for 2 or more years, and 195 patients received H-OFS for less than 2 years. The 5-year recurrence risks were not significantly lower in AI-OFS vs TAM (HR, 0.76; 95% CI, 0.38-1.33) or T-OFS vs TAM (HR, 0.87; 95% CI, 0.50-1.45) groups. Patients receiving H-OFS for 2 or more years had significantly better 5-year recurrence-free survival compared with those receiving H-OFS for less than 2 years (HR, 0.69; 95% CI, 0.54-0.90).

**Conclusions and Relevance:**

This study found no significant reductions in recurrence risk for AI-OFS and T-OFS compared with TAM alone. H-OFS duration for at least 2 years was associated with significantly improved recurrence-free survival.

## Key Points

**Question:**

Is adjuvant ovarian function suppression (OFS) associated with similar benefits in patients with premenopausal, hormone receptor–positive breast cancer in routine practice vs randomized settings?

**Findings:**

This cohort study of 2647 individuals with premenopausal, early-stage breast cancer diagnoses found no significant recurrence risk reductions among patients adding OFS to tamoxifen or aromatase inhibitor compared with tamoxifen alone. A 2-year duration of OFS was associated with reduced recurrence risk.

**Meaning:**

This study may provide insights on the use of adjuvant OFS; broader adoption of OFS in practice requires future studies to ascertain treatment outcomes and inform decision-making.

## Teaser

This cohort study investigates the association of ovarian function suppression with recurrence-free survival in routine practice vs randomized settings in patients with premenopausal hormone receptor–positive breast cancer.

## Introduction

Approximately three-quarters of breast cancers express estrogen or progesterone receptors or both; these cancers are collectively known as hormone receptor (HR)–positive disease and indicate the use of adjuvant endocrine treatment.^1^ The Suppression of Ovarian Function Trial (SOFT) and Tamoxifen and Exemestane Trial (TEXT)^2^ were designed to investigate the value of adding ovarian function suppression (OFS) to tamoxifen (TAM) and exemestane.

In 2018, combined analyses of data from SOFT and TEXT demonstrated higher rates of 8-year disease-free survival among patients receiving TAM + OFS (T-OFS) and exemestane + OFS (E-OFS) compared with patients receiving TAM alone.^3^ The most recent analyses, published in 2022, showed that 13-year disease-free survival rates were significantly higher in the E-OFS group compared with the T-OFS group (HR, 0.79; 95% CI, 0.70-0.90).^4^ From these results, guidelines recommend that TAM alone remain the standard of care in women who are premenopausal and at low risk of relapse.^5,6,7^ In women at higher risk of relapse, the combination of OFS and TAM or an aromatase inhibitor (AI) is the preferred option.^5,6,7^

Currently, little data exist on whether T-OFS or E-OFS are associated with similar benefits in clinical practice as in SOFT and TEXT.^8^ Such investigations are important for several reasons. First, many patients diagnosed with breast cancer are underrepresented in clinical trials of adjuvant systemic treatment based on young age, minority race or ethnicity, and the presence of comorbidities.^9^ Second, studies in 2020^10^and 2019^11^ showed little agreement between observational analyses for comparative efficacy research and their analogous trials in oncology, likely due to methodological limitations, including uncontrolled time-varying confounding effects, selection biases, and improper or vague definitions of treatments and follow-up periods. Causal inference frameworks in epidemiology resulted in a growing body of literature that showed that observational data may be leveraged to replicate results of a clinical trial through explicit emulation of the target trial.^12^ This approach, called target trial emulation (TTE), has been used within various health care settings.^13,14,15,16,17,18^

With updated guidelines, there is a need for studies investigating the use of OFS in observational settings. To address this gap, the TTE framework was applied to a large population-based cohort of patients who were premenopausal with HR-positive breast cancer in Alberta, Canada, with 3 research aims: to describe treatment initiation and duration patterns of adjuvant hormone treatment and OFS, compare recurrence-free survival (RFS) among 3 treatment groups (adjuvant T-OFS, AI + OFS [AI-OFS], and TAM alone), and estimate the association between duration of adjuvant OFS therapy and RFS.

## Methods

This cohort study was designed in accordance with the TTE framework.^12^ Reporting of this study follows the Strengthening the Reporting of Observational Studies in Epidemiology (STROBE) reporting guideline. This study was approved by the Health Research Ethics Board of Alberta Cancer Committee. The ethics committee waived informed consent because this was an analysis of an existing administrative database in which there was no direct contact with patients.

### Study Population and Data Sources

This population-based, retrospective cohort included all adult females aged 18 years or older diagnosed with nonmetastatic, invasive breast cancer identified through the Alberta Cancer Registry from January 1, 2010, to December 31, 2020, with no malignant neoplasm 5 years prior to diagnosis. Variables of interest were merged from the cancer registry, electronic health records, administrative claims, and vital statistics. Data were linked by each patient’s unique lifetime identifier and anonymized prior to analyses. The hospital discharge abstract database and national ambulatory care reporting system database were used to identify the presence of comorbidities and treatment-related toxic effects using *International Statistical Classification of Diseases, Tenth Revision, Clinical Modification* (*ICD-10-CM*) codes. The administrative end of follow-up in this study was April 15, 2022.

### Eligibility Criteria

We used 2 different cohorts to describe treatment patterns (aim 1) and estimate treatment outcomes (aims 2 and 3). The first cohort, called the clinical cohort, included all premenopausal females aged 18 years or older with histologically confirmed and resected HR-positive breast cancer without oophorectomy prior to breast cancer diagnosis. Given that data were not available on estradiol levels to confirm menopausal status, we defined premenopausal status as being younger than age 51 years, the mean age of menopause in Alberta.^19,20^ Patients treated with AI, including exemestane, letrozole, and anastrozole, without ovarian suppression were not considered to be premenopausal owing to contraindication. Eligibility criteria of the second cohort, called the target trial cohort, were modeled after SOFT and TEXT.^2^ Criteria from these trials and how we emulated them in our observational data are included in eTable 1 in Supplement 1. The target trial cohort included all patients from the clinical cohort who initiated TAM or AI within 12 months of surgery.

### Treatment Regimens

For aim 2, we compared patients who were adherent to the following regimens initiated at baseline for 2 years: adjuvant TAM alone vs T-OFS vs AI-OFS. This is similar to a per-protocol analysis in a randomized clinical trial, which estimates the intention-to-treat (ITT) effect in the per-protocol population. OFS treatments included gonadotropin-releasing hormone analogues goserelin (Zoladex) and leuprolide (Lupron) and bilateral surgical oophorectomy. TAM or AI must have been initiated within 12 months of surgery. Furthermore, the OFS component must have been initiated within 10 months of TAM or AI. Goserelin and leuprolide were administrated in 1- or 3-month intervals, whereas TAM and AIs were administered daily. Deviation from protocol included discontinuation of the TAM/AI or OFS component of treatment within 2 years of initiation or loss to follow-up. Therefore, patients were adherent if they continued receiving treatment for at least 2 years or developed the outcome while receiving treatment within this period.

For aim 3, we compared patients with a 2-year or greater duration of hormone therapy (TAM or AI) + OFS (H-OFS) vs those with a less than 2-year duration. Treatment duration was defined as the date of hormone treatment initiation to the earliest of date of discontinuing the TAM/AI or OFS component of treatment, date of last follow-up, or date of recurrence or death. Patients could receive any other neoadjuvant or adjuvant chemotherapy, human epidermal growth factor receptor 2 (ERBB2, also known as HER2)–targeted therapy, and radiation therapy.

### Outcomes of Interest, Time Zero, and Follow-Up Period

For aim 1, outcomes of interest were time to treatment initiation, measured from date of surgery to first treatment, and treatment duration, measured from date of first treatment receipt to date of last receipt. The primary outcome of interest for aims 2 and 3 was 5-year RFS. The occurrence of relapses is not routinely collected in administrative data; thus, a case-finding algorithm by Jung et al^21^ was used to identify patients with a recurrence. Time zero or baseline was defined as the date of hormone treatment initiation. Patients were followed up until recurrence, death, reaching study end after 5 years, censoring due to administrative end of follow-up, or censoring due to protocol deviation, whichever occurred first. Overall survival was not investigated owing to limited follow-up time and events in young patients.

### Statistical Analysis

All patient variables were described using means, medians, SDs, and IQRs for numeric variables and frequency tables with proportions for categorical variables. The Kaplan-Meier method was used to estimate survival curves for time to treatment initiation, treatment duration, and median times with 95% CIs.

For aim 2, patients were censored for protocol deviations, introducing the possibility of time-varying selection bias through informative censoring. We used a Cox proportional hazards model to estimate hazard ratios (HRs) and 95% CIs with time-fixed inverse probability of treatment weights (IPTWs) to adjust for baseline confounding and time-varying inverse probability of censoring weights (IPCWs) to adjust for bias from censoring. An IPTW is defined as the inverse of an individual’s probability of initiating their respective treatment, conditional on time-fixed covariates, and estimated using multinomial logistic regression. An IPCW is defined as the inverse of an individual’s probability of remaining uncensored at each point, conditional on their time-fixed and varying covariates and estimated using pooled logistic regression. The weight of an individual at each time point was the product of their IPTW and IPCW, truncated at the 99th percentile and not stabilized.

The analysis plan for aim 3 involved 3 stages previously described to address immortal time bias.^18,22^ First, participants were cloned, and a copy was assigned to each treatment strategy. Second, copies were artificially censored when they deviated from their assigned treatment strategy. For example, copies assigned to a 2-year or greater duration of H-OFS were censored if they discontinued within 2 years. Lastly, time-varying IPCWs were used to address selection bias due to artificial censoring of clones, and a weighted Cox model was used to estimate HRs and 95% CIs. IPCWs for aim 3 were also estimated using pooled logistic regression.

Time-fixed covariates included age, treatment facility, tumor stage, number of positive lymph nodes, tumor grade, type of breast surgery, type of lymph node surgery, and receipt of chemotherapy, anti-ERBB2 therapy, and radiation therapy. Time-varying covariates included incidence of severe toxic effects, number of incident minor toxic effects, and number of clinical visits during follow-up. Severe toxic effects included cardiovascular diseases, cerebrovascular diseases, and thrombosis and embolisms. Minor toxic effects included all remaining toxic effects present in eTable 2 in Supplement 1. These toxic effects were based on SOFT and TEXT and studies that defined treatment-associated toxic effects in observational data using *ICD-10-CM* codes.^23,24^

In all analyses, IPTWs and IPCWs were calculated at 3-month intervals to emulate how pharmaceutical OFS is administered in practice. All tests were 2-sided, and statistical significance was defined by *P* < .05. Analyses were performed in RStudio statistical software version 2022.07 (RStudio). Data were analyzed from July 2022 through March 2023.

## Results

**Figure 1.**
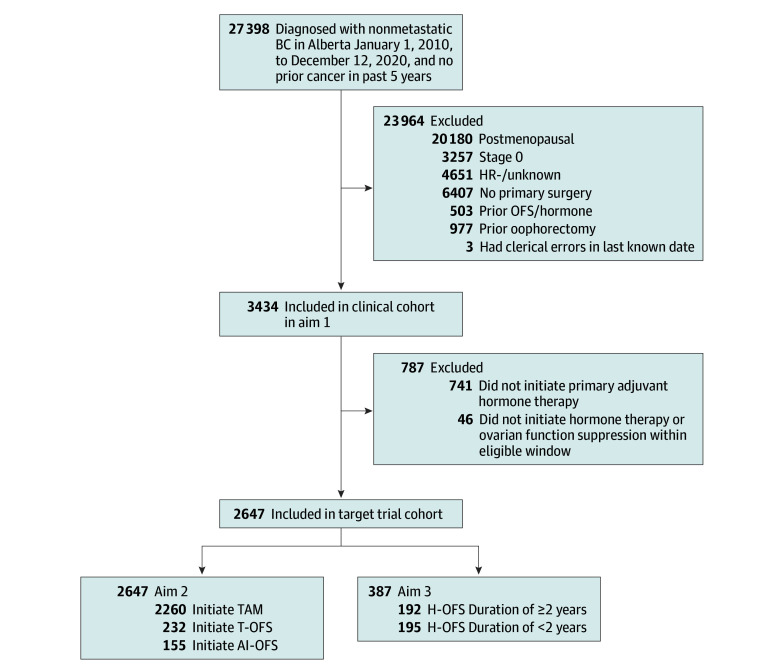
Study Flowchart Patient inclusion and exclusion in clinical and target trial cohorts based on eligibility criteria are presented. AI-OFS indicates aromatase inhibitor plus OFS; BC, breast cancer; H-OFS, hormone treatment plus OFS; HR, hormone receptor; OFS, ovarian function suppression; TAM, tamoxifen; T-OFS, TAM plus OFS.

In total, 3434 patients (median [IQR] age, 45 [40-48] years; 3434 female [100%]) were premenopausal with resected HR-positive breast cancer and included in our clinical cohort (Figure 1). Most patients were ERBB2 negative and node negative, with T1 tumors. In the clinical cohort, 505 patients (14.7%) initiated OFS and 696 patients (20.3%) underwent oophorectomy. Complete patient characteristics of the clinical cohort are described in eTable 3 in Supplement 1. Table 1 presents treatment initiation and duration in the clinical cohort. The median time to hormone therapy initiation was 127.5 days (95% CI, 122.0-133.0 days), and 2611 of 2696 individuals (96.9%) who initiated hormone therapy did so within 9 months of surgery. The median time to OFS initiation was 316.0 days (95% CI, 277.0-384.0 days) from surgery and 164.0 days (95% CI, 122.0-215.0 days) from hormone therapy initiation; 285 of 498 patients initiating OFS (57.2% ) began within 9 months of starting hormone therapy. For oophorectomy, the median time to treatment was 771.5 days (95% CI, 690.0-841.0 days) from surgery and 611.5 days (95% CI, 546.0-677.0 days) from hormone therapy initiation. The median duration of hormone therapy was greater than that of OFS in 2010 to 2014 (622.0 days [95% CI, 547.0-764.0 days] vs 335.4 days [95% CI, 272.3-549.3 days]), but the median duration was similar in 2015 to 2019 (Table 1). Finally, the number of patients concurrently initiating OFS and hormone therapy notably increased after 2014, as did the number of patients receiving AI (eFigure in Supplement 1).

**Table 1.** Patterns of Treatment Initiation and Duration

	Time, median (95% CI), d	Patients, No. (%)^a^				
		3 mo	At 6 mo	At 9 mo	At 12 mo	At 2 y
**Time to treatment initiation**						
Surgery to treatment initiation						
Hormone therapy (n = 2696)	127.5 (122.0-133.0)	1126 (41.8)	2065 (76.6)	2611 (96.9)	2650 (98.3)	2664 (98.9)
OFS (n = 505)	316.0 (277.0-384.0)	78 (15.4)	149 (29.5)	226 (44.8)	269 (53.3)	342 (67.7)
Oophorectomy (n = 696)	771.5 (690.0-841.0)	12 (1.7)	35 (5.0)	87 (12.5)	154 (22.1)	337 (48.4)
Hormone initiation to OFS initiation						
OFS (n = 498)	164.0 (122.0-215.0)	200 (40.2)	252 (50.6)	285 (57.2)	307 (61.6)	366 (73.5)
Oophorectomy (n = 630)	611.5 (546.0-677.0)	46 (7.3)	94 (14.9)	143 (22.7)	202 (32.1)	352 (55.9)
**Duration of treatment**						
2010-2014						
Hormone therapy (n = 1264)	622.0 (547.0-764.0)	1082 (85.5)	973 (75.7)	825 (65.3)	742 (58.7)	603 (47.9)
OFS (n = 157)	335.4 (272.3-549.3)	139 (88.5)	109 (69.4)	88 (56.1)	76 (48.4)	56 (35.7)
2015-2019						
Hormone therapy (n = 1431)	477.0 (463.0-525.0)	1143 (79.7)	1001 (69.5)	838 (58.5)	773 (54.0)	539 (37.6)
OFS (n = 348)	487.4 (426.3-610.3)	316 (90.8)	273 (78.4)	242 (69.5)	205 (58.9)	142 (40.8)

Abbreviations: OFS, ovarian function suppression.

^a^ Percentages are out of row totals. Numbers and percentages are for patients initiating treatment for time to treatment initiation and patients receiving treatment for treatment duration.

Of 3434 patients in the clinical cohort, 2647 individuals satisfied eligibility criteria for inclusion in the target trial cohort to estimate the association of remaining adherent to AI-OFS vs T-OFS vs TAM alone for 2 years and the association of an H-OFS duration of 2 years or greater vs less than 2 years with 5-year RFS (Figure 1). In total, 2260 patients (85.4%) initiated TAM alone and 888 patients were adherent to protocol; 232 patients (8.7%) initiated T-OFS and 144 individuals were adherent, while 155 patients (5.9%) initiated AI-OFS and 108 individuals were adherent. Furthermore, 192 patients received H-OFS for 2 or more years and 195 individuals received it for less than 2 years. Significant differences between patients initiating TAM, T-OFS, and AI-OFS were observed for tumor stage, number of positive lymph nodes, tumor grade, breast surgery type, and receipt of chemotherapy and radiation therapy (Table 2). Univariable comparisons between patients with an H-OFS duration of 2 years or greater vs less than 2 years did not reveal significant differences for any variable (Table 2).

**Table 2.** Baseline Characteristics of Patients Who Satisfied Target Trial Eligibility Criteria

Characteristic	Patients initiating treatment, No. (%)			*P* value	Patients receiving H-OFS, No. (%)		*P* value
	TAM alone (n = 2260)	T-OFS n = 232)	AI-OFS (n = 155)		Duration <2 y (n = 195)	Duration ≥2 y (n = 192)	
Age, mean (SD)	43.6 (5.88)	41.2 (5.66)	42.9 (5.48)	<.001	41.7 (6.08)	41.9 (5.26)	.68
Treatment zone							
Urban	1990 (88.1)	211 (90.9)	130 (83.9)	.11	172 (88.2)	169 (88.0)	>.99
Not urban	270 (11.9)	21 (9.1)	25 (16.1)		23 (11.8)	23 (12.0)	
ER status							
Negative	34 (1.5)	2 (0.9)	1 (0.6)	.83	1 (0.5)	2 (1.0)	.98
Positive	2225 (98.5)	230 (99.1)	154 (99.4)		194 (99.5)	190 (99.0)	
Missing	1 (0.0)	0	0		0	0	
PR status							
Negative	281 (12.4)	36 (15.5)	22 (14.2)	.66	32 (16.4)	26 (13.5)	.47
Positive	1977 (87.5)	196 (84.5)	133 (85.8)		163 (83.6)	166 (86.5)	
Missing	2 (0.1)	0	0		0	0	
ERBB2 (also known as HER2) status							
Negative	1822 (80.6)	191 (82.3)	121 (78.1)	.58	156 (80.0)	156 (81.3)	.14
Positive	417 (18.5)	40 (17.2)	31 (20.0)		35 (17.9)	36 (18.8)	
Missing	21 (0.9)	1 (0.4)	3 (1.9)		4 (2.1)	0	
T stage							
T1	1186 (52.5)	80 (34.5)	54 (34.8)	<.001	75 (38.5)	59 (30.7)	.07
T2	879 (38.9)	111 (47.8)	71 (45.8)		82 (42.1)	100 (52.1)	
T3	162 (7.2)	33 (14.2)	24 (15.5)		28 (14.4)	29 (15.1)	
T4	33 (1.5)	8 (3.4)	6 (3.9)		10 (5.1)	4 (2.1)	
No. of positive lymph nodes							
0	1361 (60.2)	102 (44.0)	44 (28.4)	<.001	81 (41.5)	65 (33.9)	.22
1-3	767 (33.9)	114 (49.1)	82 (52.9)		95 (48.7)	101 (52.6)	
≥4	132 (5.8)	16 (6.9)	29 (18.7)		19 (9.7)	26 (13.5)	
Grade							
I	294 (13.0)	14 (6.0)	9 (5.8)	<.001	14 (7.2)	9 (4.7)	.33
II	942 (41.7)	86 (37.1)	50 (32.3)		61 (31.3)	75 (39.1)	
III	981 (43.4)	111 (47.8)	77 (49.7)		101 (51.8)	87 (45.3)	
Missing	43 (1.9)	21 (9.1)	19 (12.3)		19 (9.7)	21 (10.9)	
Charlson Comorbidity Index score							
0	2117 (93.7)	214 (92.2)	139 (89.7)	.12	174 (89.2)	179 (93.2)	.19
≥1	143 (6.3)	18 (7.8)	16 (10.3)		21 (10.8)	13 (6.8)	
Chemotherapy							
No	760 (33.6)	38 (16.4)	19 (12.3)	<.001	30 (15.4)	27 (14.1)	.87
Yes	1500 (66.4)	194 (83.6)	136 (87.7)		165 (84.6)	165 (85.9)	
Anti-ERBB2 therapy							
No	1891 (83.7)	192 (82.8)	126 (81.3)	.71	160 (82.1)	158 (82.3)	>.99
Yes	369 (16.3)	40 (17.2)	29 (18.7)		35 (17.9)	34 (17.7)	
Radiation							
No	659 (29.2)	50 (21.6)	31 (20.0)	.004	45 (23.1)	36 (18.8)	.34
Yes	1601 (70.8)	182 (78.4)	124 (80.0)		150 (76.9)	156 (81.3)	
Breast surgery type							
BCS	1059 (46.9)	79 (34.1)	69 (44.5)	<.001	81 (41.5)	67 (34.9)	.26
Mastectomy	1067 (47.2)	139 (59.9)	85 (54.8)		105 (53.8)	119 (62.0)	
Missing	134 (5.9)	14 (6.0)	1 (0.6)		9 (4.6)	6 (3.1)	
Lymph node surgery type							
SLNB	1343 (59.4)	139 (59.9)	89 (57.4)	.27	115 (59.0)	113 (58.9)	.95
ALND	683 (30.2)	76 (32.8)	57 (36.8)		66 (33.8)	67 (35.1)	
Missing	234 (10.4)	17 (7.3)	9 (5.8)		14 (7.2)	12 (6.3)	

Abbreviations: AI-OFS, aromatase inhibitor plus OFS; ALND, axillary lymph node dissection; BCS, breast conserving surgery; ER, estrogen receptor; ERBB2, human epidermal growth factor receptor 2; H-OFS, hormone treatment plus OFS; OFS, ovarian function suppression; PR, progesterone receptor; SD, standard deviation; SLNB, sentinel lymph node biopsy; T, tumor; TAM, tamoxifen; T-OFS, TAM plus OFS.

**Figure 2.**
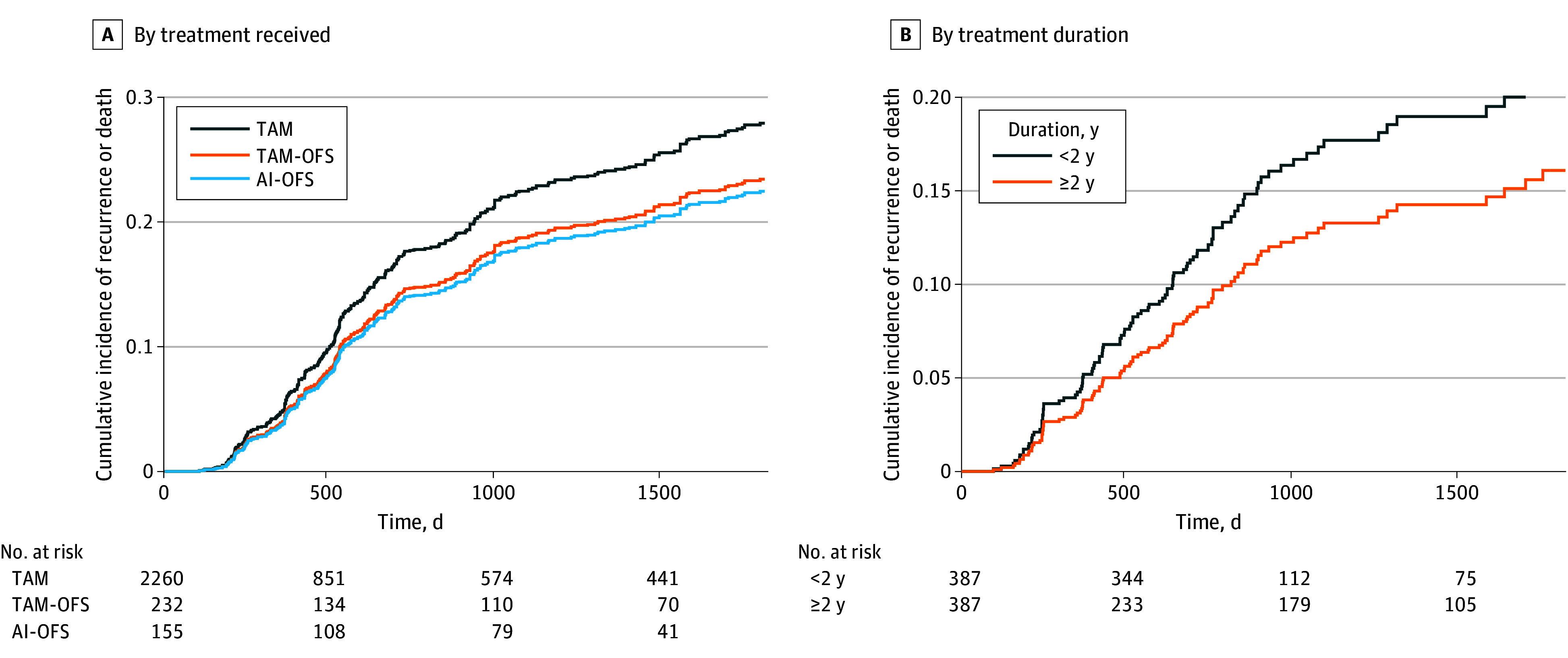
Cumulative Incidence of Recurrence or Death Cumulative incidence curves of recurrence or death estimated from Cox proportional hazards models are presented for A) patients adherent to treatment for 2 years, stratified by treatment received (tamoxifen [TAM] alone, tamoxifen with ovarian function suppression [TAM-OFS], and aromatase inhibitor with ovarian function suppression [AI-OFS]) and B, for patients receiving hormone therapy (TAM or AI) and OFS, stratified by a treatment duration of less than 2 years or 2 years or more.

Table 3 demonstrates association estimates comparing recurrence hazard between TAM, T-OFS, and AI-OFS. In the overall population, the estimated event rates at 5 years from the weighted Cox model were 27.7% (95% CI, 23.6 %-31.2%), 23.3% (95% CI, 18.0%-36.6%), and 21.4% (95% CI, 11.8%-33.5%) in TAM, T-OFS, and AI-OFS groups, respectively (Figure 2A). The hazard of recurrence was not significantly lower in the AI-OFS group compared with the TAM group (HR, 0.76; 95% CI, 0.38-1.33) or in the T-OFS group compared with the TAM group (HR, 0.87; 95% CI, 0.50-1.45). Similar hazards of recurrence were observed in the subgroup of patients who received neoadjuvant or adjuvant chemotherapy (Table 3).

**Table 3.** Estimates of Recurrence Risk by Baseline 2-y Treatment

Subgroup	Treatment type	HR (95% CI)	*P* value
Overall^a^	TAM	1 [Reference]	NA
	T-OFS	0.87 (0.50-1.45)	.51
	AI-OFS	0.76 (0.38-1.33)	.36
Prior chemotherapy	TAM	1 [Reference]	NA
	T-OFS	0.88 (0.62-1.65)	.53
	AI-OFS	0.73 (0.44-1.72)	.43
Age <40 y	TAM	1 [Reference]	NA
	T-OFS	0.55 (0.16-1.83)	.24
	AI-OFS	0.63 (0.16-1.99)	.30
Age ≥40 y	TAM	1 [Reference]	NA
	T-OFS	0.95 (0.55-2.61)	.69
	AI-OFS	0.89 (0.50-2.74)	.78
ERBB2 (also known as HER2) positive	TAM	1 [Reference]	NA
	T-OFS	0.83 (0.21-2.98)	.53
	AI-OFS	0.57 (0.16-2.12)	.48
ERBB2 negative	TAM	1 [Reference]	NA
	T-OFS	0.84 (0.45-1.75)	.60
	AI-OFS	0.76 (0.40-1.59)	.54
Lymph node positive	TAM	1 [Reference]	NA
	T-OFS	0.86 (0.54-2.05)	.61
	AI-OFS	0.84 (0.67-2.04)	.56
Lymph node negative	TAM	1 [Reference]	NA
	T-OFS	0.88 (0.60-2.10)	.76
	AI-OFS	0.76 (0.25-2.13)	.63
T1	TAM	1 [Reference]	NA
	T-OFS	0.78 (0.25-1.93)	.47
	AI-OFS	0.82 (0.27-2.22)	.51
T2-4	TAM	1 [Reference]	NA
	T-OFS	0.86 (0.47-1.59)	.64
	AI-OFS	0.61 (0.29-1.26)	.18
Grade I-II	TAM	1 [Reference]	NA
	T-OFS	0.96 (0.47-2.66)	.87
	AI-OFS	0.75 (0.37-1.78)	.58
Grade III	TAM	1 [Reference]	NA
	T-OFS	0.87 (0.45-1.92)	.84
	AI-OFS	0.58 (0.23-1.39)	.23

Abbreviations: AI-OFS, aromatase inhibitor plus OFS; ERBB2, human epidermal growth factor receptor 2; HR, hazard ratio; NA, not applicable; OFS, ovarian function suppression; T, tumor; TAM, tamoxifen; T-OFS, tamoxifen plus OFS.

^a^ The *P* value for the test of the proportional hazards assumption = .68.

The hazard of recurrence was 31% lower among patients with an H-OFS duration of 2 years or greater compared with patients receiving H-OFS for less than 2 years (HR, 0.69; 95% CI, 0.54-0.90) (eTable 4 in Supplement 1). The estimated event rates at 5 years from the weighted Cox model were 16.1% (95% CI, 12.7%-19.3%) for the 2-year and greater duration and 22.3% (95% CI, 19.5%-26.1%) for the less than 2-year duration groups (Figure 2B). Similar relative reductions were observed for most subgroups (eTable 4 in Supplement 1).

## Discussion

To our knowledge, this cohort study is the first study to describe OFS patterns and outcomes in Alberta, Canada. Hormone therapy and OFS were not initiated simultaneously, and more than half of individuals initiating OFS (285 individuals [57.2%]) began 9 months after starting hormone therapy. In total, 14.7% of patients in the clinical cohort initiated OFS and 20.3% underwent oophorectomy. The prevalence and median time to oophorectomy were likely overestimated given that we could not confirm whether oophorectomy procedures were received with the intent of treatment. We also observed increased initiation of OFS after 2014, coinciding with the publications of SOFT and TEXT. Using an insurance claims database, Reeder-Hayes et al^25^ described increasing OFS use in the US, with a mean prevalence of 11.3% in 2016. A multicenter cohort study in Portugal^26^ showed higher prevalence rates, with a mean of 15.5% in 2006 to 2013, increasing to 25% in 2014 to 2015. The combination of OFS with TAM was preferred in our cohort, although OFS with AI is becoming more common.^26^ Few studies have assessed clinical adherence to OFS. Reeder-Hayes et al^25^ showed similar adherence to H-OFS and hormone therapy alone, with a median duration of 2.5 years. Differences in treatment duration between H-OFS and hormone therapy alone were not observed after 2014, although median duration was shorter in our study.

To our knowledge, this is also the first study to apply TTE for the effectiveness of OFS treatment in premenopausal, HR-positive breast cancer. Patients initiating T-OFS and AI-OFS were more likely to have a higher tumor and nodal stage, have a higher grade, and receive mastectomy, chemotherapy, and radiation therapy. Our analysis demonstrated no significant recurrence risk reductions for AI-OFS or T-OFS vs TAM in the overall cohort or subgroups. SOFT and TEXT demonstrated greater recurrence risk reductions for AI-OFS and T-OFS groups in subgroups with node-negative tumors and tumors 2 cm or less in size compared with subgroups with node-positive tumors and tumors greater than 2 cm in size. Furthermore, these trials showed more favorable outcomes in T-OFS groups compared with AI-OFS in the ERBB2-positive subgroup.

These discrepancies are likely associated with the relatively higher baseline recurrence risk for patients in our clinical cohort compared with SOFT and TEXT. A higher proportion of patients in this study were T2 to T4, ERBB2 positive, node positive, and grade III and received chemotherapy than SOFT and TEXT populations. This likely resulted in a different distribution of prognostic variables within subgroups such that similar heterogenous outcomes were not observed, particularly in the ERBB2-positive subgroup. Furthermore, we emulated a per-protocol analysis, whereas SOFT and TEXT reported ITT effects. The ITT effect is an estimate of being assigned to a treatment group with no consideration for treatment adherence. However, there is no guarantee that the effect of assigned treatment represents the effectiveness in routine practice.^27^ This study specified a 2-year protocol owing to recent implementation of OFS in Alberta and few patients completing 5 years of T-OFS and AI-OFS, which may explain attenuated estimates and failure to achieve statistical significance. The 2020 Addition of Ovarian Suppression to Tamoxifen in Young Women With Hormone-Sensitive Breast Cancer Who Remain Premenopausal or Regain Vaginal Bleeding After Chemotherapy (ASTRRA) trial^28^ showed significantly improved disease-free survival among 635 South Korean patients aged 45 years or younger who added 2 years of OFS to 5 years of TAM compared with TAM alone, with notable benefits in patients aged younger than 35 years. These findings suggest that the benefit of a 2-year OFS regimen is likely pronounced by completing 5 years of TAM compared with 2 years.

Significant reductions in recurrence risk were observed among patients receiving H-OFS for 2 or more years compared with less than 2 years in the overall cohort and most subgroups. This adds to increasing evidence highlighting the importance of improving the methodological rigor of studies assessing the effect of treatment duration and the use of cloning and IPCWs. Studies comparing duration of treatment are prone to immortal time bias.^12,22,29^ In our study, assigning recurrence outcomes within the first 2 years of follow-up among patients still receiving treatment to the group with a less than 2-year duration would result in outcome misclassification. This would make the group with a 2-year or greater duration immortal for the first 2 years of follow-up and exaggerate the protective outcome associated with a 2-year or greater duration in H-OFS therapy.^22,29^

### Limitations

This study demonstrates several limitations of examining OFS outcomes in observational data. The first is that toxic effects were captured through the hospital discharge abstract database and national ambulatory care reporting system database, which indicate sufficient severity to warrant a hospital visit. To account for minor toxic effects, counts of clinical visits were used. However, we did not account for toxic effects that did not result in a hospital or clinical visit but could be associated with treatment adherence, resulting in potential residual confounding. Clinical visits may also have been unrelated to treatment-related toxic effects. The second limitation was lack of access to patient estrogen levels to define menopausal status and recovery from chemotherapy-induced amenorrhea. Although we used similar surrogates as in previous studies,^26,28,30^ postmenopausal individuals may have been included in the group receiving TAM alone and bias association estimates toward the null. This may be particularly true for patients aged 40 years and older. A third limitation was that we did not have a definitive date of recurrence. Patients would not have been captured by the outcome algorithm if they were not treated for their recurrence, further reducing study power. In a fourth limitation, most patients receiving OFS in our cohort received chemotherapy, so we could not estimate outcomes in patients without prior chemotherapy. Given the disease-free survival benefit of E-OFS in this subgroup of SOFT and TEXT, this may help answer whether AI-OFS can provide sufficient prognosis without chemotherapy in patients who are not clearly high risk.

## Conclusions

The broader adoption of OFS in practice requires comparative effectiveness studies to ascertain that trial benefits are observed in routine practice and inform decision-making. This cohort study applied TTE to patients who were premenopausal, with HR-positive breast cancer to estimate the effectiveness of adding OFS to adjuvant endocrine therapy. Modeling eligibility criteria and treatment definitions of our observational study from SOFT and TEXT helped approximate similar point estimates and directions of effect. The addition of OFS to adjuvant endocrine therapy was indicated for patients at high risk in our cohort, but the initiation of OFS did not consistently occur within similar windows as proposed by SOFT and TEXT. As more data become available, future observational studies should estimate treatments outcomes at 10 and 15 years and explore reasons for delays in treatment and early discontinuation.

## Notes

Supplement 1. **eTable 1.** Eligibility Criteria for Inclusion in Target Trial Based on Clinical Trial Eligibility Criteria **eTable 2.** Description of *ICD-10-CM* Codes Used to Define Treatment-Related Toxic Effects **eTable 3.** Baseline Characteristics of Patients With Premenopausal Hormone Receptor–Positive Breast Cancer in Alberta 2010-2020 Who Underwent Primary Surgery and No Prior Oophorectomy **eTable 4.** Estimates of Recurrence Risk by Duration of Hormone Treatment With Ovarian Function Suppression **eFigure 1.** Patterns of Concurrent Ovarian Function Suppression and Tamoxifen or Aromatase Inhibitor Initiation Over Time

Supplement 2.
Data Sharing Statement
