# Supplementary material for: Adjuvant Ovarian Function Suppression in Premenopausal Hormone Receptor–Positive Breast Cancer
Source: JAMA Netw Open. 2024 Mar 13;7(3):e242082. doi: 10.1001/jamanetworkopen.2024.2082 (PMC10938175; doi:10.1001/jamanetworkopen.2024.2082)
Supplement: Supplement 1. — eTable 1. Eligibility Criteria for Inclusion in Target Trial Based on Clinical Trial Eligibility Criteria eTable 2. Description of ICD-10-CM Codes Used to Define Treatment-Related Toxic Effects eTable 3. Baseline Characteristics of Patients With Premenopausal Hormone Receptor–Positive Breast Cancer in Alberta 2010-2020 Who Underwent Primary Surgery and No Prior Oophorectomy eTable 4. Estimates of Recurrence Risk by Duration of Hormone Treatment With Ovarian Function Suppression eFigure 1. Patterns of Concurrent Ovarian Function Suppression and Tamoxifen or Aromatase Inhibitor Initiation Over Time [file jamanetwopen-e242082-s001.pdf]

Supplemental Online Content

Basmadjian RB, Lupichuk S, Xu Y, Quan ML, Cheung WY, Brenner DR. Adjuvant ovarian function suppression in premenopausal hormone receptor–positive breast cancer. *JAMA Netw Open*. 2024;7(3):e242082.
doi:10.1001/jamanetworkopen.2024.2082

eTable 1. Eligibility Criteria for Inclusion in Target Trial Based on Clinical Trial Eligibility Criteria
eTable 2. Description of *ICD-10-CM* Codes Used to Define Treatment-Related Toxic Effects
eTable 3. Baseline Characteristics of Patients With Premenopausal Hormone Receptor–Positive Breast Cancer in Alberta 2010-2020 Who Underwent Primary Surgery and No Prior Oophorectomy
eTable 4. Estimates of Recurrence Risk by Duration of Hormone Treatment With Ovarian Function Suppression
eFigure. Patterns of Concurrent Ovarian Function Suppression and Tamoxifen or Aromatase Inhibitor Initiation Over Time

This supplemental material has been provided by the authors to give readers additional information about their work.

© 2024 Basmadjian RB et al. *JAMA Network Open.*

eTable 1. Eligibility criteria for inclusion in the target trial based on SOFT/TEXT eligibility criteria.

SOFT/TEXT	Trial emulation
**Inclusion criteria**	
• Premenopausal women based on estradiol (E_2) levels in the premenopausal range (according to institutional parameters).	• Premenopausal women based on age <51 years.
• Histologically proven, resected breast cancer. Proper surgery for primary breast cancer includes a total mastectomy or breast conserving procedure.	• Same.
• Hormone receptor positive tumors.	• Same.
• Tumour must be confined to the breast and axillary nodes without detected metastases elsewhere.	• Same.
**Exclusion criteria**	
• Women who are postmenopausal (i.e., do not have an estradiol (E2) level in the premenopausal range) after surgery or after chemotherapy, whichever is later.	• Postmenopausal women based on age ≥51 years or receipt of an aromatase inhibitor without ovarian function suppression.
• Distant metastatic disease.	• Same.
• Locally advanced inoperable breast cancer including inflammatory breast cancer or supraclavicular node involvement or with enlarged internal mammary nodes (unless pathologically negative) are not eligible.	• All patients who did not undergo primary surgery in our cohort were not included.
• Patients with positive final margins (referring to only DCIS and invasive cancer, not LCIS).	• Not emulated.

© 2024 Basmadjian RB et al. *JAMA Network Open.*

- History of prior ipsilateral or contralateral invasive breast cancer, or other previous or concomitant invasive malignancy.

- Same.

- Patients with other non-malignant systemic diseases that would prevent prolonged follow-up.

- Patients with psychiatric, addictive, or any disorder that would prevent compliance with protocol requirements.

**Comorbid condition**	**ICD-10-CM code**
Myocardial infarction	I21, I22, I252
Congestive heart failure	I099, I110, I130, I132, I255, I420, I425-I429, I43, I50, P290
Unstable angina	I200
Complicated hypertension	I11-I13, I15, I16
Pulmonary embolism	I26
Cerebrovascular disease	G45, G46, I60-I69, H340
Moderate or severe liver diseases	I850, I859, I864, I982, K704, K711, K721, K729, K765, K766, K767
Renal disease and renal failure	I120, I13.1, N032-N037, N052-N057, N18, N19, N25, Z490, Z492, Z940, Z992
Chronic pulmonary disease	I278, I279, J40-J47, J60-J67, J684, J701, J703
Interstitial lung disease	J84
Pneumonitis	R0602
Diabetes with chronic complications	E102-E105, E107, E112-E115, E117, E122-E125, E127, E132-E135, E137, E142-E145, E147
Peptic ulcer disease	K25-K28
Dementia/Alzheimer's	F00-F03, F051, G30, G311
Schizophrenia	F20, F22, F25, F30-39
Alcohol and drug abuse	F10, E52, G621, I426, K292, K700, K703, K709, T51, Z502, Z714, Z721, F11-F16, F18, F19, Z715, Z722
Intellectual disability	F70-F79

© 2024 Basmadjian RB et al. *JAMA Network Open.*

• Patients with a history of noncompliance to medical regimens and patients who are considered potentially unreliable.	• Not emulated.
• Patients who have had a prior bilateral oophorectomy or ovarian irradiation, or who have received GnRH analogues as part of their breast cancer treatment prior to randomization.	• Same.
• Patients who are pregnant or lactating at the time of randomization or who desire a pregnancy within 5 years.	• Not emulated.
• Patients who were taking tamoxifen or other SERM (e.g., Raloxifene) or hormone replacement therapy (HRT) within one year prior to their breast cancer diagnosis. Prior oral contraceptives are allowed.	• Same.

SOFT = Suppression of Ovarian Function Trial; TEXT = Tamoxifen and Exemestane Trial

© 2024 Basmadjian RB et al. *JAMA Network Open.*

eTable 2. Description of ICD-10-CM codes used to define treatment-related toxic effects.

**Targeted Adverse Events in SOFT/TEXT**	**Data available from DAD/NACRS**	**ICD-10-CM code to emulate treatment-related toxicity**
Cardiac infarction or ischemia	Cardiovascular diseases, not including thrombosis/embolisms, cerebrovascular diseases, and hypertensive disease	Rheumatic heart diseases: I00-I09<br>Ischemic heart diseases: I20-I25<br>Pulmonary heart disease and diseases of pulmonary circulation: I27.1, I27.8, 127.9, I28<br>Other forms of heart disease: I30-I5A<br>Diseases of arteries, arterioles, and capillaries: I70-I73, I77-I79<br>Diseases of veins, lymphatic vessels, and lymph nodes, not elsewhere classified: I83-I89<br>Symptoms and signs involving the circulatory and respiratory systems: R00-R01<br>Syncope and collapse: R55
CNS hemorrhage or ischemia	Cerebrovascular diseases	Cerebrovascular diseases: I60-I69<br>Transient cerebral ischemic attacks and related syndromes: G45<br>Vascular syndromes of brain in cerebrovascular diseases: G46
Thrombosis or embolism	Thrombosis and embolisms	Pulmonary heart disease and diseases of pulmonary circulation: I26<br>Diseases of arteries, arterioles, and capillaries: I74-177<br>Diseases of veins, lymphatic vessels, and lymph nodes, not elsewhere classified: I80-I82
Glucose intolerance (diabetes)	Diabetes mellitus and other glucose disorders	Drug or chemical induced diabetes mellitus: E09<br>Type 1 diabetes mellitus: E10<br>Type 2 diabetes mellitus: E11<br>Other specified diabetes mellitus: E13<br>Nondiabetic hypoglycemic coma: E15<br>Other disorders of pancreatic internal secretion: E16.0-E16.3
Hyperglycemia		

© 2024 Basmadjian RB et al. *JAMA Network Open.*

		Postprocedural hypoinsulinemia: E89.1<br>Abnormal findings on examination of blood – abnormal glucose level: R73
Hypertension	Hypertensive diseases	Hypertensive diseases: I10-I16<br>Hypotension: I95<br>Primary pulmonary hypertension: I27.0<br>Other secondary pulmonary hypertension: I27.2<br>Chronic venous hypertension: I87.3<br>Abnormal blood-pressure reading, without diagnosis: R03
Fatigue	Malaise and fatigue	Malaise and fatigue: R53<br>Other specified nonpsychotic mental disorders: F48.8
Nausea	Nausea	Nausea and vomiting: R11
Sweating	Hyperhidrosis	Generalized hyperhidrosis: R61<br>Focal hyperhidrosis: L74.5
Insomnia	Insomnia and sleep disorders	Insomnia: G47.0<br>Insomnia not due to a substance or known physiological condition: F51.0<br>Problems related to sleep: Z72.82<br>Insufficient sleep syndrome: F51.12
Depression	Mood (affective) disorders	Depressive episode: F32<br>Major depressive disorder: F33
Musculoskeletal symptoms	Diseases of musculoskeletal and connective tissue	Inflammatory polyarthropathies: M05-M14<br>Osteoarthritis: M15-M19<br>Other joint disorders: M20-M25<br>Dorsopathies and spondylopathies: M45-M48.3, M48.8-M54<br>Disorders of muscles and tendons: M60-M67<br>Disorders of bone density and structure: M80-M83, M85-M90<br>Disorders of cartilage: M91-M94

Osteoporosis		Other and unspecified soft tissue disorders, not elsewhere classified: M79<br>Pain in throat and chest: R07<br>Abdominal and pelvic pain: R10<br>Pain, unspecified: R52<br>Pain, not elsewhere classified: G89<br>Migraine and other headache syndromes: G43-G44<br>Headache: R51
Bone fracture	Injuries and other consequences of external causes	Fracture of skull and facial bones: S02<br>Fracture of cervical vertebra and other parts of neck: S12<br>Fractur of ribs, sternum, and thoracic spine: S22<br>Fracture of lumbar spine and pelvis: S32<br>Fracture of shoulder and upper arm: S42:<br>Fracture of forearm: S52<br>Fracture at wrist and hand level: S62<br>Fracture of femur: S72<br>Fracture of lower leg and ankle: S82<br>Fracture of foot and toe: S92<br>Fatigue fracture of vertebra: M48.4<br>Collapsed vertebra, not elsewhere classified: M48.5<br>Disorder of continuity of bone: M84
Vaginal dryness<br>Dyspareunia<br>Urinary incontinence	Diseases of genitourinary system	Other specified urinary incontinence: N39.4<br>Stress incontinence: N39.3<br>Unspecified urinary incontinence: R32<br>Functional urinary incontinence: R39.81<br>Inflammatory diseases of female pelvic organs: N70-N77

© 2024 Basmadjian RB et al. *JAMA Network Open.*

		Noninflammatory disorders of female genital tract: N80-N98
Decreased libido	Libido and sexual desire	Decreased libido: R68.82<br>Hypoactive sexual desire disorder: F52.0
Allergic reaction/hypersensitivity		Allergy, unspecified: T78.4<br>Anaphylactic reaction due to adverse effect of correct drug or medicament properly administered: T88.6<br>Unspecified adverse effect of drug or medicament: T88.7
Hot flushes	Fever	Fever of other and unknown origin: R50

© 2024 Basmadjian RB et al. *JAMA Network Open.*

eTable 3. Baseline characteristics of patients with premenopausal hormone receptor positive breast cancer in Alberta from 2010 to 2020 who underwent primary surgery and no prior oophorectomy.

	Total (N=3434)
**Follow-up time**	
Mean (SD)	2530 (1010)
Median [IQR]	2430 (1674, 3392)
**Age at Dx**	
Mean (SD)	43.7 (5.81)
Median [Min, Max]	45.0 [40.0, 48.0]
**Treatment zone, N (%)**	
Urban	3009 (87.6%)
Rural	425 (12.4%)
**ER status, N (%)**	
ER-	58 (1.7%)
ER+	3375 (98.3%)
Missing	1 (0.0%)
**PR status, N (%)**	
PR-	450 (13.1%)
PR+	2981 (86.8%)
Missing	3 (0.1%)
**Her2 status, N (%)**	
HER2-	2771 (80.7%)
HER2+	609 (17.7%)
Missing	54 (1.6%)
**T stage, N (%)**	
T1	1852 (53.9%)
T2	1265 (36.8%)
T3	250 (7.3%)
T4	67 (2.0%)
**N stage, N (%)**	
N0	2063 (60.1%)
N1	1080 (31.5%)
N2	205 (6.0%)
N3	86 (2.5%)
**Number of positive lymph nodes, N (%)**	
Zero	2104 (61.3%)
1 to 3	1026 (29.9%)
4+	304 (8.9%)
**Grade, N (%)**	

© 2024 Basmadjian RB et al. *JAMA Network Open.*

I	534 (15.6%)
II	1362 (39.7%)
III	1414 (41.2%)
Missing	124 (3.6%)
**Charlson comorbidities index, N (%)**	
Zero	3177 (92.5%)
>=1	257 (7.5%)
**Surgery type, N (%)**	
BCS	1633 (47.6%)
Mastectomy	1599 (46.6%)
Missing	202 (5.9%)
**Lymph node surgery type, N (%)**	
SLNB	1986 (57.8%)
ALND	1071 (31.1%)
Missing	377 (11.0%)
**Chemotherapy, N (%)**	
No chemotherapy	1328 (38.7%)
Chemotherapy	2106 (61.3%)
**Anti-HER2 therapy, N (%)**	
No anti-HER2	2924 (85.1%)
Immunotherapy	510 (14.9%)
**Radiation, N (%)**	
No radiation	1048 (30.5%)
Radiation	2386 (69.5%)
**Hormone therapy, N (%)**	
No hormone therapy	739 (21.5%)
Hormone therapy	2695 (78.5%)
**OFS Treatment, N (%)**	
No OFS	2929 (85.3%)
OFS	505 (14.7%)
**Ovarian treatment, N (%)**	
Neither	2403 (70.0%)
Oophorectomy only	526 (15.3%)
OFS only	335 (9.8%)
OFS+ oophorectomy	170 (5.0%)

Abbreviations: ALND = axillary lymph node dissection; BCS = breast conserving surgery; ER = estrogen receptor; HER2 = human epidermal growth factor receptor 2; OFS = ovarian function suppression; PR = progesterone receptor; SD = standard deviation; SLNB = sentinel lymph node biopsy; T = tumour.

© 2024 Basmadjian RB et al. *JAMA Network Open.*

eTable 4. Estimates of Recurrence Risk by Duration of Hormone Treatment With Ovarian Function Suppression

**Subgroup**	**H-OFS treatment duration, y**	**HR (95% CI)**	***P* value**
Overall[a]	<2	1 [Reference]	NA
	≥2	0.69 (0.54-0.90)	.005
Prior chemotherapy	<2	1 [Reference]	NA
	≥2	0.68 (0.52-0.91)	.005
Age <40 y	<2	1 [Reference]	NA
	≥2	0.67 (0.42-1.08)	.09
Age ≥40 y	<2	1 [Reference]	NA
	≥2	0.72 (0.52-0.99)	.04
ERBB2 (also known as HER2) positive	<2	1 [Reference]	NA
	≥2	0.58 (0.28-1.20)	.14
ERBB2 negative	<2	1 [Reference]	NA
	≥2	0.77 (0.57-1.04)	.09
Lymph node positive	<2	1 [Reference]	NA
	≥2	0.72 (0.52-0.99)	.04
Lymph node negative	<2	1 [Reference]	NA
	≥2	0.79 (0.51-1.20)	.32
T1	<2	1 [Reference]	NA
	≥2	0.86 (0.51-1.48)	.60
T2-4	<2	1 [Reference]	NA
	≥2	0.67 (0.47-0.96)	.03
Grade I-II	<2	1 [Reference]	NA
	≥2	0.86 (0.58-1.29)	.48
Grade III	<2	1 [Reference]	NA
	≥2	0.65 (0.44-0.97)	.03

Abbreviations: ERBB2, human epidermal growth factor receptor 2; H-OFS, hormone treatment with ovarian function suppression; HR, hazard ratio; NA, not applicable; T, tumor.
[a]The P value for the test of the proportional hazards assumption = .18.

© 2024 Basmadjian RB et al. *JAMA Network Open.*

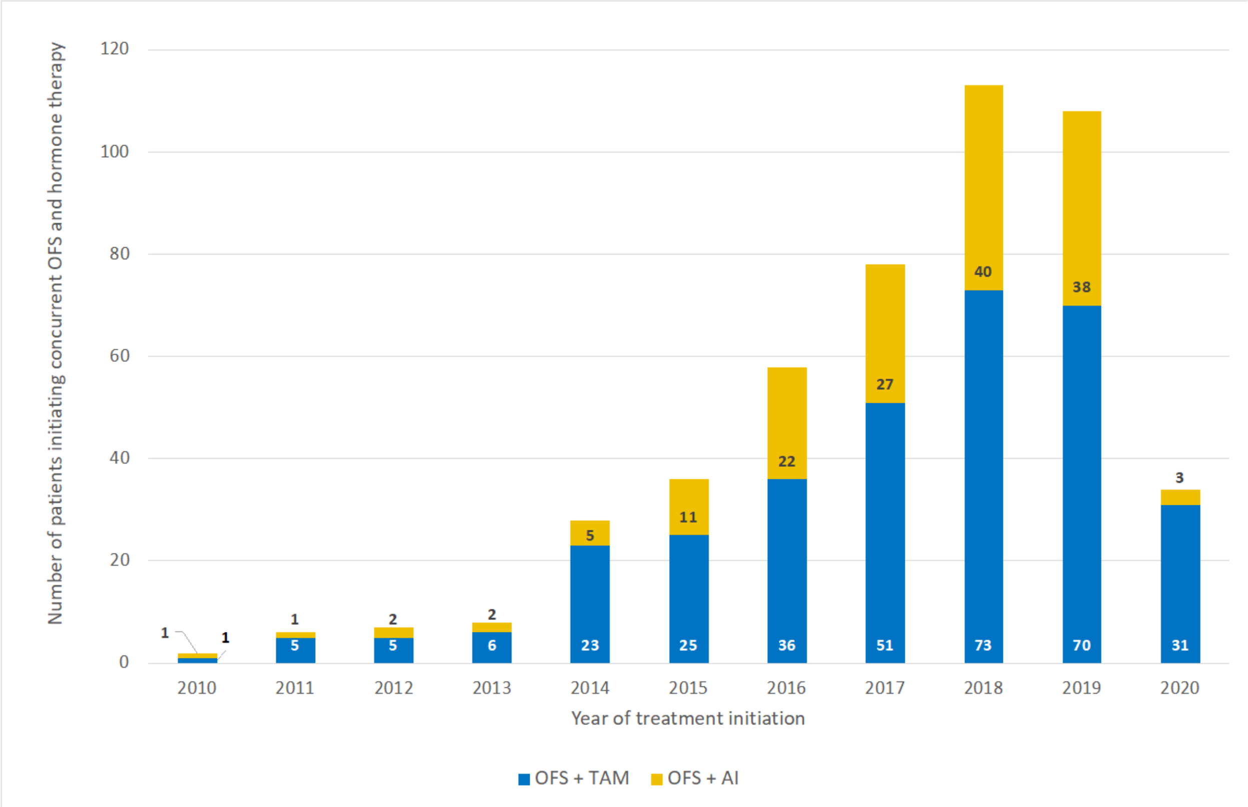

eFigure 1. Patterns of concurrent ovarian function suppression and tamoxifen or aromatase inhibitor initiation over time. Abbreviation: AI = aromatase inhibitor; OFS = ovarian function suppression, TAM = tamoxifen.

© 2024 Basmadjian RB et al. *JAMA Network Open.*
