# Supplementary material for: Adjuvant Ovarian Function Suppression in Premenopausal Hormone Receptor–Positive Breast Cancer
Source: JAMA Netw Open. 2024 Mar 13;7(3):e242082. doi: 10.1001/jamanetworkopen.2024.2082 (PMC10938175; doi:10.1001/jamanetworkopen.2024.2082)
Supplement: Supplement 2. — Data Sharing Statement [file jamanetwopen-e242082-s002.pdf]

## Data Sharing Statement

Basmadjian. Adjuvant Ovarian Function Suppression in Premenopausal Hormone Receptor–Positive Breast Cancer. *JAMA Netw Open*. Published online March 13, 2024. doi:10.1001/jamanetworkopen.2024.2082

**Data**
**Data available:** No
